# *Bacillus subtilis* PcrA Couples DNA Replication, Transcription, Recombination and Segregation

**DOI:** 10.3389/fmolb.2020.00140

**Published:** 2020-07-21

**Authors:** María Moreno-del Alamo, Rubén Torres, Candela Manfredi, José A. Ruiz-Masó, Gloria del Solar, Juan Carlos Alonso

**Affiliations:** ^1^Department of Microbial Biotechnology, Centro Nacional de Biotecnología, CNB-CSIC, Madrid, Spain; ^2^Centro de Investigaciones Biológicas Margarita Salas, CIB-CSIC, Madrid, Spain

**Keywords:** replication fork stalling, RNA polymerase backtracking, replication-transcription conflict, RecL16, Rep, UvrD

## Abstract

*Bacillus subtilis* PcrA abrogates replication-transcription conflicts *in vivo* and disrupts RecA nucleoprotein filaments *in vitro*. Inactivation of *pcrA* is lethal. We show that PcrA depletion lethality is suppressed by *recJ* (involved in end resection), *recA* (the recombinase), or *mfd* (transcription-coupled repair) inactivation, but not by inactivating end resection (*addAB* or *recQ*), positive and negative RecA modulators (*rarA* or *recX* and *recU*), or genes involved in the reactivation of a stalled RNA polymerase (*recD*2, *helD, hepA*, and *ywqA*). We also report that *B. subtilis* mutations previously designated as *recL16* actually map to the *recO* locus, and confirm that PcrA depletion lethality is suppressed by *recO* inactivation. The *pcrA* gene is epistatic to *recA* or *mfd*, but it is not epistatic to *addAB, recJ, recQ, recO16, rarA, recX, recU, recD*2, *helD, hepA*, or *ywqA* in response to DNA damage. PcrA depletion led to the accumulation of unsegregated chromosomes, and this defect is increased by *recQ, rarA*, or *recU* inactivation. We propose that PcrA, which is crucial to maintain cell viability, is involved in different DNA transactions.

## Introduction

Homologous recombination is the major pathway to circumvent a replicative stress, a replication fork collapse and for the elimination of DNA double-strand breaks (DSBs) induced by endogenous or exogenous stress. Super-family 1 (SF1) DNA helicases, which are conserved motor proteins that couple nucleoside triphosphate hydrolysis to the unwinding of duplex DNA, play crucial roles in repair-by-recombination and in coping with replication-transcription conflicts (RTCs) (Wu and Hickson, 2006; Singleton et al., 2007). The prototype of bacterial SF1 helicases that translocate with 3′ → 5′ direction is UvrD (Singleton et al., 2007; Dillingham, 2011). This enzyme shares a significant degree of structural similarity with Rep, which is restricted to the γ-Proteobacteria Class, PcrA and yeast Srs2 DNA helicases (Wu and Hickson, 2006; Marini and Krejci, 2010). Rep and PcrA play essential roles in the replication of extrachromosomal elements, whereas UvrD and PcrA participate in the resolution of RTCs by poorly understood mechanisms (Boubakri et al., 2010; Bruning et al., 2014; Epshtein et al., 2014; Merrikh et al., 2015). *In vitro* studies reveal that PcrA and UvrD interact with the RNA polymerase (RNAP), Rep interacts with the replicative DNA helicase (DnaB in Proteobacteria) and Srs2 physically interacts with Rad51 and with the PCNA sliding clamp (ortholog of bacterial DnaN) among other proteins (Antony et al., 2009; Guy et al., 2009; Kaniecki et al., 2017; Sanders et al., 2017). Absence of *Escherichia coli* Rep and UvrD renders cells inviable when grown in rich medium (Taucher-Scholtz et al., 1983), but lack of *Bacillus subtilis* PcrA renders cells inviable even when grown in minimal medium (Petit et al., 1998; Merrikh et al., 2015). To gain insight into the crucial steps carried on by PcrA, a comparative analysis with UvrD and Rep was undertaken. From the comparative analysis of *E. coli* (best-characterized representative of the Proteobacteria Phylum) and *B. subtilis* (best-characterized from the Firmicutes Phylum), which are evolutionarily separated by more than 2,000 million years, a genetic divergence larger than that between human and paramecium, we expect to understand the role of the SF1 UvrD-like DNA helicases.

When the DNA of a single inert mature haploid non-replicating chromosome of a *B. subtilis* spore is damaged, the RTCs of the single spore genome are compounded during the rapid outgrowth. In the absence of end resection (AddAB and/or RecJ-RecQ [RecS]) cells remain recombination proficient and apparently are as capable of repairing damaged template bases as the wild type (*wt*) control (Vlasic et al., 2014). These spores, which lack an intact homologous template, require RecA mediators (RecO, RecR), RecA itself, and positive (RecF) and negative (RecX, RecU) RecA modulators, with PcrA facilitating DNA replication through transcription units (Vlasic et al., 2014; Merrikh et al., 2015; Raguse et al., 2017). It is likely that PcrA is implicated in the processing of damaged replication forks and/or recombination intermediates formed at damaged forks and in circumventing DNA lesions in the absence of an intact homologous template, without generating a fork breakage that should be lethal for the revival of a haploid spore (Raguse et al., 2017). Then, a nucleoprotein RecA filament aids to overcome RTCs *via* different DNA damage tolerance pathways and to reactivate replication by recruiting the damage checkpoint DisA and pre-primosome DnaD proteins (Million-Weaver et al., 2015; Torres et al., 2019). This is consistent with the observation that the lethality of *B. subtilis* Δ*pcrA* cells is suppressed by *recF*17, *recL*16, Δ*recO*, or Δ*recR* mutations (Petit and Ehrlich, 2002).

In *E. coli* cells, the synthetic lethality of Δ*uvrD* Δ*rep* is partially suppressed by *recJ, recQ, recO, recR*, or *recF* inactivation in minimal medium, but it is only marginally suppressed by *recA* inactivation (Lestini and Michel, 2008; Guy et al., 2009). Inactivation of *recJ* or *recQ* provides very limited suppression of Δ*rep* Δ*uvrD* rich medium lethality, but *recA* inactivation does not (Petit and Ehrlich, 2002; Veaute et al., 2005; Lestini and Michel, 2008; Guy et al., 2009). *in vivo* assays reveal that PcrA expression in *E. coli* can substitute several functions of UvrD, but antagonizes the function of Rep, providing a heterologous dominant negative phenotype (Petit et al., 1998). These data suggest that in the absence of UvrD and Rep, toxic RecA nucleoprotein filaments and/or RecA-mediated recombination intermediates or DNA structures can accumulate. This hypothesis is supported by the following observations: (i) in budding yeast, suppressors of Srs2 mutations map in the Rad51 gene (Aboussekhra et al., 1992); and (ii) RecA- or Rad51-mediated DNA strand exchange is actively prevented by the PcrA, UvrD, or Srs2 DNA helicase *in vitro*, considered as a paradigmatic anti-recombinase activity (Krejci et al., 2003; Veaute et al., 2003, 2005; Anand et al., 2007; Park et al., 2010; Fagerburg et al., 2012; Petrova et al., 2015; Kaniecki et al., 2017).

Other studies have demonstrated that the rich-medium synthetic lethality of Δ*uvrD* Δ*rep* cells is caused primarily by RTCs, with partial reduction of transcription or translation rates across heavily transcribed genes in the opposite orientation relative to the replication forks, to compensate the impact of transcription on DNA replication (Guy et al., 2009; Baharoglu et al., 2010; Kamarthapu et al., 2016; Myka et al., 2017). Indeed, rich-medium synthetic lethality of Δ*uvrD* Δ*rep* cells is fully suppressed by reducing transcription, as mutations in different RNAP subunits (rpo^*^ [*rpoB* and *rpoC* point mutants]), or reducing translation elongation, such as by mutations in a tRNA gene (AspRS, *aspT*), in an aminoacyl tRNA synthetase, in a translation factor needed for efficient formation of proline-proline bonds (EF-P), and *spoT*1 mutation [encoding a (p)ppGpp pyrophosphorylase-defective SpoT] (Guy et al., 2009; Baharoglu et al., 2010; Kamarthapu et al., 2016; Myka et al., 2017). Transcription and translation are coupled with the leading ribosome pushing RNAP forward; however, when these two processes become uncoupled, RNAP rpo^*^ mutants are prone to pausing with (p)ppGpp promoting UvrD-mediated RNAP backtracking (Kamarthapu et al., 2016; Myka et al., 2017). These observations altogether open the question about the primary cause of PcrA lethality. (Unless stated otherwise, indicated genes and products are of *B. subtilis* origin. The nomenclature used to denote the origin of proteins from other bacteria is based on the bacterial genus and species abbreviation [*e.g., E. coli* UvrD is referred to as UvrD_*Eco*_]).

The physiological causes of PcrA/UvrD-Rep_*Eco*_ lethality is/are poorly understood. To understand the role of PcrA in rich medium exponentially growing *B. subtilis* cells, we have studied the genetic linkage of PcrA depletion (Merrikh et al., 2015) with mutations in genes acting at the presynaptic (Δ*recJ*, Δ*recQ*, Δ*addAB, recL*16, Δ*rarA*, Δ*recX*, Δ*recU*) and synaptic (Δ*recA*) stages, as well as in genes that contribute to bypass RTCs or facilitate RNAP backtracking or removal (Δ*helD*, Δ*recD*2 [absent in *E. coli*], Δ*hepA* [also termed *yqhH*or *rapA*], Δ*ywqA* or Δ*mfd*). We show that PcrA depletion reduced cell viability by >4,000-fold, and survival in the presence of limiting H_2_O_2_ or methyl methanesulfonate (MMS) concentrations, suggesting that PcrA is involved in repair-by-recombination. The *recL*16 mutations were mapped in the *recO* gene and the mutation was termed *recO*16. The PcrA depletion lethality is suppressed by *recO16, recJ, recA*, or *mfd* inactivation, but not by *recQ, rarA, recX, recU, addAB, helD, hepA*, or *ywqA* inactivation. The *pcrA* gene is not epistatic to *recO16, recJ, rarA, recQ, recX, recU, recD*2, *addAB, helD, hepA*, or *ywqA* in response to MMS-induced DNA damage, but it is epistatic to *recA* or *mfd*. Absence of PcrA promotes a net accumulation of unsegregated nucleoids, and this defect is increased in the absence of RarA, RecQ, and RecU. We conclude that PcrA contributes to untangle branched intermediates and works at the interface of DNA replication, transcription, recombination and segregation.

## Materials and Methods

### Bacterial Strains and Plasmids

All *B. subtilis* strains used derived from BG214, and are listed in Table 1. The gene to be characterized was deleted by gene replacement with the *six*-*cat*-*six* (SCS) cassette flanked by appropriate homologous regions up- and downstream. The SCS cassette, composed of two directly oriented β-recombinase cognate sites (*six* sites) and the *cat* gene, confers chloramphenicol resistance (Cm^R^) (Rojo and Alonso, 1995). Natural competent cells were transformed with the SCS cassette flanked by homologous regions to the gene to be deleted with selection for Cm^R^. Integration of the SCS cassette, through double crossover recombination, replaced the gene under characterization. The β recombinase promoter, which maps within the six site, can read the downstream gene to overcome any potential polar effect (Rojo et al., 1994). A plasmid-borne β-recombinase gene was moved into the background, and followed by β site-specific recombinase-mediated excision between the two directly oriented six sites, leading to the deletion of the *cat* gene and one six site (Rojo et al., 1994). The final outcome of this strategy is that the gene to be characterized is replaced by a single six site (Sanchez et al., 2005). Accuracy of deletions was confirmed by complementation with a plasmid-borne gene (Sanchez et al., 2005). The IPTG-inducible *sspB* cassette encoding the SspB adaptor protein (a gift from Houra and Christopher Merrikh, Vanderbilt University, USA) was ectopically integrated into the *amy* locus by natural transformation (Alonso et al., 1988; Torres et al., 2017). The null *hepA* or *ywqA* mutations (a gift from Marie-Agnès Petit, Université Paris-Saclay, France) were moved into the BG214 background (Table 1) by SPP1-mediated transduction (Valero-Rello et al., 2017).

**Table 1:** *Bacillus subtilis* strains.

**Strains^a^**	**Relevant genotype**	**Source/references**
BG214	Wild type	Laboratory strain
BG107^b^	+ *recL*16 *hem*E *pks*L *dea*D	Alonso et al., 1987
BG107-1	+ *recO*16	This work
BG1873	+ Δ*recA*	This work
BG763	+ Δ*recJ*	Sanchez et al., 2006
BG705	+ Δ*recQ*	Sanchez et al., 2006
BG633	+ Δ*recU*	Fernández et al., 1998
BG1065	+ Δ*recX*	Cárdenas et al., 2012
BG551	+ Δ*helD*	Carrasco et al., 2001
BG1067	+ Δ*rarA*	Romero et al., 2019b
BG1887	+ Δ*mfd*	This work
BG1779	+ Δ*recD*2	Torres et al., 2017
BG1337	+ Δ*addAB*	Torres et al., 2017
BG1525	+ *pcrA-ssrA sspB*	Torres et al., 2017
BG1583	+ *pcrA-ssrA sspB* Δ*recD*2	Torres et al., 2017
BG1861	+ Δ*hepA* (also termed *yqhH*)	This work
BG1862	+ Δ*ywqA*	This work
BG1715	+ *pcrA-ssrA sspB recO*16	This work
BG1877	+ *pcrA-ssrA sspB* Δ*recA*	This work
BG1731	+ *pcrA-ssrA sspB* Δ*recJ*	This work
BG1713	+ *pcrA-ssrA sspB* Δ*recQ*	This work
BG1709	+ *pcrA-ssrA sspB* Δ*recX*	This work
BG1711	+ *pcrA-ssrA sspB* Δ*recU*	This work
BG1823	+ *pcrA-ssrA sspB* Δ*rarA*	This work
BG1869	+ *pcrA-ssrA sspB* Δ*addAB*	This work
BG1859	+ *pcrA-ssrA sspB* Δ*helD*	This work
BG1875	+ *pcrA-ssrA sspB* Δ*mfd*	This work
BG1839	+ *pcrA-ssrA sspB* Δ*hepA*	This work
BG1857	+ *pcrA-ssrA sspB* Δ*ywqA*	This work
Plasmid	Relevant genotype	Source/reference
pQE1-pcrA	AmpR, *ori_*Eco*_*	This work
pHP14	CmR, EmR *ori_*Eco*_ ori_*Bsu*_*	de la Hoz et al., 2000
pCB1133	pHP14 + *pcrA* K37A	This work
pCB1119	pHP14 + *pcrA* Q254A (E224V)	This work
pCB1225	pHP14 + *pcrA* T65I (pcrA3)	This work

a *All strains are derivatives of B. subtilis BG214 (trpCE metA5 amyE1 ytsJ1 rsbV37 xre1 xkdA1 att^SP^ att^ICEBs1^) strain*.

b *The phenotype of the original BG107 strain is defined elsewhere (C.M., M.C.G., M.M.C. and J.C.A.) (Supplementary Material, Annex 1)*.

The *ssrA* degradation tag fused to the 3′-end of the *pcrA* gene (*pcrA*-*ssrA*) replaces the *pcrA* gene (Merrikh et al., 2015; Torres et al., 2017). The *pcrA*-*ssrA* gene was moved by SPP1-mediated transduction into *recO*16 *sspB*, Δ*recJ sspB*, Δ*recD*2 *sspB*, Δ*recQ sspB*, Δ*recX sspB*, Δ*recU sspB*, Δ*rarA sspB*, Δ*addAB sspB*, Δ*helD sspB*, Δ*hepA sspB*, Δ*ywqA sspB*, or Δ*mfd sspB* strain as well as in the otherwise *wt* background (*rec*^+^ *sspB* strain). The Δ*recA* mutation was mobilized by SPP1-mediated transduction into the *pcrA*-*ssrA sspB* (*pcrA*_T_) context.

To segregate the *recO* mutations and to reconstruct a new BG107 strain (BG107-1), limiting concentrations of chromosomal DNA of the original BG107 (*recL*16) strain and 0.1 μg/ml of pHP14 DNA were added to competent BG214 cells, with selection for the plasmid-borne Cm^R^ marker. From those transformants it was expected that by co-transformation of any unlinked markers (congression) 0.1 to 1% of the Cm^R^ cells should receive the *recL*16 MMS sensitive (MMS^S^) phenotype (Alonso et al., 1988). Selection of MMS^S^ was performed by streaking colonies on agar plate containing or lacking MMS (Alonso et al., 1988). Five selected MMS^S^ clones were sequenced by high-throughput sequence analyzer (Illumina) technology using standard sequencing libraries and filtered sequence data (Beijing Genomics Institute [BGI]), of ~1 gigabases per sample, followed by whole-genome comparison with the nucleotide sequencing of the MMS^R^ parental BG214 strain.

The K37A, T65I, or Q254A *pcrA* variants were generated by means of mutation site directed mutagenesis (QuickChange Kit, Stratagene) using the pQE-1-borne *wt pcrA* plasmid as a template. Unexpectedly, all the Q254A mutants analyzed also contained the unselected E224V mutation. K37A, T65I, or Q254A-E224V *pcrA* genes were amplified by PCR and were cloned into *Xma*I-*Bam*HI-cleaved pHP14. The resultant recombinant plasmids were used to transform competent *B. subtilis* BG214 cells.

### Survival Assays and Colony Size

Plating exponentially growing *pcrA*-*ssrA sspB* (*pcrA*_T_) cells in rich medium onto agar plates containing isopropyl-β-D thiogalactopyranoside (IPTG) induced SspB expression from a regulated promoter, which then bound the SsrA peptide tag and rapidly delivered the tagged PcrA-SsrA protein to the *B. subtilis* ClpXP protease for degradation (PcrA degron [*pcrA*_T_] strain) (Keiler et al., 1996; Griffith and Grossman, 2008; Merrikh et al., 2015). PcrA degron cultures were grown to OD_560_ = 0.4. The cultures were divided and aliquots plated in LB agar plates alone or with 500 μM IPTG (Calbiochem). The percentage of colony forming units (CFUs) in LB agar plates containing IPTG was measured. The mean and SEM were calculated using Prism 6 software (GraphPad), and a Student's *t*-test, with *P* < 0.01, was performed to denote the threshold of significance.

Cell sensitivity to chronic MMS (Sigma Aldrich) or H_2_O_2_ (Sigma Aldrich) exposure was determined by growing cultures to OD_560_ = 0.4 and plating appropriate dilutions on rich LB agar plates containing IPTG (500 μM) and MMS (1.3 mM) or H_2_O_2_ (0.2 mM) as described (Sánchez et al., 2007). Cells grew in rich LB medium with a doubling time of 28–35 min. Plates were incubated overnight (16–18 h, 37°C) and the number of CFUs determined. Experiments were conducted independently at least four times. Fractional survival data are shown as mean ± SEM. Statistical analysis was performed with a two-tailed Student's *t*-test. For experiments involving more than two groups, one-way analysis of variance (ANOVA) was performed. For all tests, a *P* < 0.1 was considered significant (Supplementary Material). All statistical analyses were performed using Prism 6 software.

*B. subtilis* cells form round smooth colonies that raised above the agar. Colony size on Petri dishes was calculated via the diameter of a hypothetical circular colony. After overnight incubation, Petri dishes pictures were acquired and analyzed with the aid of a BioRad ChemiDoc^TM^ imaging system equipped with the QuantityOne software (BioRad). The relative mean colony diameter of ~50 isolated blind scoring colonies from *pcrA*_T_ vs. *pcrA*_T_ cells bearing a second mutation were measured from the pictures using ImageJ software (NIH). Upon colony magnification, the relative mean colony size was calculated using the formula for the area of a circle. Average and standard deviation were calculated using Prism 6 software. The colony area was compared by analysis of variance or Student's *t*-test, with *P* < 0.01 as the threshold of significance.

### Nucleotide Sequence Analysis

The samples of genomic DNA from *B. subtilis wt* (BG214, Reference strain) and the Test *recL16* (BG107) strains were analyzed using the first step of comparative genome sequencing, a service provided by NimbleGen Systems, Inc. as described earlier (Albert et al., 2005). Briefly, the genome of *B. subtilis wt* was tiled on custom-designed “mapping” microarrays with 29 base oligonucleotide pieces (probes) and a 7 or 8 base spacing from the start of one probe to the start of the next. The genomic DNA from the Reference BG214 strain was labeled with Cy5 and of the Test BG107 strain with Cy3. The ratios of hybridization intensities (reference/test) were calculated and plotted against genome position. If the test sample contains no mutation, the reference/test ratio equals 1. This analysis gives a high-resolution map of possible mutation sites, in which each mutation is localized in a window of 29 bases (the length of the reporting probe). A custom algorithm, based on hybridization intensity ratios significantly above background that have good agreement between results from corresponding probes from both strands, allows the identification of likely sites of mutation. The data were analyzed graphically using the SignalMap software provided by NimbleGen. The regions around the probes with a reference/test ratio significantly >1 were tested for mutations by direct DNA sequencing. Sequencing was performed with Big Dye (from Operon Technologies Inc.) using the protocols of the University of Wisconsin Biotechnology Center, after PCR amplification of the target genomic DNA sequence.

The samples of genomic DNA from *B. subtilis wt* (BG214, Reference strain) and five MMS^S^ clones from the newly constructed. Test strain were re-sequenced by high-throughput sequence analyzer (Illumina) technology using standard sequencing libraries and filtered sequence data, a service provided by BGI, of ~1 gigabase per sample. The sequencing data were used to conduct paired-end nucleotide sequencing with the *rec*^+^ BG214 reference strain and the five MMS^S^ clones from the newly constructed BG107-1 sample as described (Quail et al., 2008).

### Fluorescence Microscopy and Data Analysis

For chromosome segregation analyses (**Figure 2**), cells were fixed and stained as described (Carrasco et al., 2004). To obtain exponentially growing cells, overnight cultures were inoculated in LB rich medium. The *recO*16, Δ*recA*, Δ*rarA*, Δ*recJ*, Δ*recQ*, Δ*recX*, or Δ*recU* mutants in the *pcrA*-*ssrA sspB* (*pcrA*_T_) context (Table 1) were grown unperturbed in LB medium to OD_560_ = 0.2 (37°C). IPTG (500 μM) was added to half of the culture, and both cultures were incubated (60 min, 37°C). Then, cells were collected, subjected to fixation with 2% formaldehyde, and finally stained with 4′,6′-diamino-2-phenylindole (DAPI) (1 μg/ml). Samples were visualized and photographed by fluorescence microscopy with a Hamamatsu 3CCD Digital Camera C7780 coupled to a BX61 Olympus fluorescence microscope, equipped with an 100x immersion oil lens and a DAPI filter (U-MNU2).

The ImageJ software (NIH) was used to merge the phase contrast and DAPI-fluorescence images, which allowed us to distinguish the septum, and thus determine the filamentation event and was also used to determine the cell length. Blind scoring was performed on captured images as described (Carrasco et al., 2004).

## Results and Discussion

### Experimental Rationale

The phenotypes associated with the absence of PcrA are exceedingly complex and reflect an involvement in several aspects of DNA metabolism, including DNA replication, transcription, RTCs, repair-by-recombination and chromosomal segregation. The PcrA depletion lethality was attributed to the accumulation of toxic RecA-mediated intermediates or SOS toxicity. In fact, inactivation of positive RecA mediators (*recO, recR*), modulator (*recF*) (Table S1) or an unknown function (*recL*) suppresses the Δ*pcrA* lethality of cells grown in minimal medium (Petit and Ehrlich, 2002). In addition, a mutation in *recO, recR, recF*, or *recL* reduces and delays the SOS induction (Gassel and Alonso, 1989). However, the lethality in the Δ*pcrA recA*1 (a leaky *recA* mutation) challenged such hypothesis (Petit and Ehrlich, 2002). This observation was revisited and extended using *rec*-deficient strains (Table 1).

PcrA-like enzymes contribute to release stalled RTCs with the subsequent recruitment of repair factors or to modulate the re-initiation of DNA replication and transcription (Guy et al., 2009; Boubakri et al., 2010; Merrikh et al., 2015). Several *B. subtilis* translocases, namely the SF1 DNA helicases (as PcrA, HelD, RecD2) and the SF2 enzymes (Mfd, HepA [also termed YqhH] and YwqA) have been shown to interact with the RNAP (Selby and Sancar, 1993; Muzzin et al., 1998; Sukhodolets et al., 2001; Shaw et al., 2008; Yawn et al., 2009; Wiedermannova et al., 2014; Sanders et al., 2017; Le et al., 2018). The poorly characterized HepA and YwqA enzymes, which belong to the Swi2/Snf2 family of translocases, share ~30% identity to *HepA*_*Eco*_. These translocases actively process a RNAP backwards as in the case of a RNAP stalled elongation complex (backtracking) or remove RNAP from the DNA template to resolve RTCs (Table S2) (Muzzin et al., 1998; Sukhodolets et al., 2001; Shaw et al., 2008; Yawn et al., 2009; Wiedermannova et al., 2014; Sanders et al., 2017; Le et al., 2018). The contribution of these functions to the inviability of PcrA depletion will be tested.

**Figure 1:**
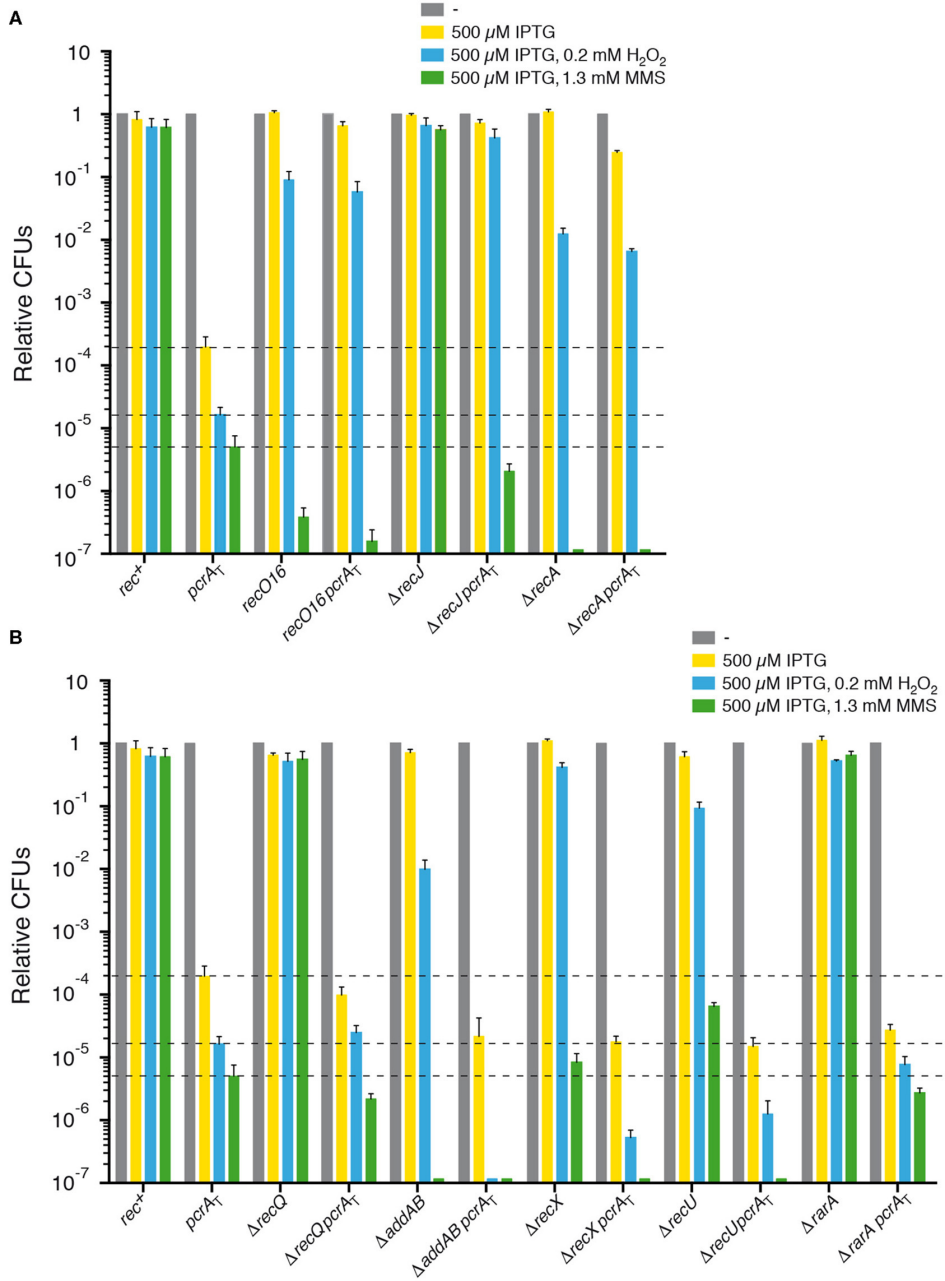
PcrA lethality is suppressed by *recJ, recO*, or *recA* inactivation, but not by *addAB, recQ, rarA, recX*, or *recU* inactivation. Log phase cultures of *wt*, single and double mutant strains were diluted and plated on LB agar containing 500 μM IPTG (yellow bars) or lacking it (gray bars). Lethality assays showing cell viability upon PcrA depletion in *recJ, recO*, or *recA* cells **(A)** and in *addAB, recQ, rarA, recX*, or *recU* cells **(B)**. Log phase cultures of indicated strains were diluted and plated on LB agar containing IPTG and 0.2 mM H_2_O_2_ (blue bars) or IPTG and 1.3 mM MMS (green bars). Experiments were performed at least four times. The dotted lines mark the survival rate upon PcrA depletion. Data are shown as mean fractional survival ± SEM.

Finally, the UvrD_*Eco*_ or PcrA helicase/translocase plays a crucial role in nucleotide excision repair (Mendonca et al., 1993; Petit and Ehrlich, 2002; Epshtein, 2015), but only the former contributes to DNA mismatch repair (Lenhart et al., 2016). In this report, we show that PcrA also contributes to the repair of non-bulky lesions of oxidative nature generated upon exposure to MMS or H_2_O_2_ (Figure 1). Upon PcrA depletion, cells were exposed to limiting MMS or H_2_O_2_ concentrations, whose lesions are specifically removed by base excision repair (Sedgwick, 2004; Fu et al., 2012). If damaged template bases escape specialized repair, because they are in ssDNA regions, the offending lesion halts DNA polymerase (DNAP) or RNAP progression, and the lesion-containing gap is circumvented *via* damage avoidance pathways (template switching, fork reversal, translesion synthesis). When the damage is bypassed and the lesion is present in duplex DNA, it is removed by specialized pathways (Sedgwick, 2004; Fu et al., 2012).

### PcrA ATP Binding Mutants Render Cells Unviable

PcrA3_*Sau*_ (T61I), defective in ATPase and helicase activities, and the PcrA_*Sau*_ (PcrAH^−^) mutant variant (K33A Q250A), which lacks ATPase and helicase activities, can displace RecA_*Eco*_ from ssDNA, and inhibit RecA_*Eco*_-mediated DNA strand exchange *in vitro* (Anand et al., 2007), suggesting that ATP binding and hydrolysis and the DNA helicase activity of PcrA are dispensable for its anti-recombinase activity. To assess whether the ATPase and/or translocase activity of *B. subtilis* PcrA are essential for cell proliferation, plasmid-borne *pcrA* genes with T65I, K37A, or Q254A mutations, equivalent to *S. aureus* mutations, were constructed and used to replace the *wt pcrA* gene. The single *pcrA* Q254A mutant gene also contained the unselected E224V mutation. The crystal structure of PcrA_*Bst*_ DNA helicase with ADP suggests that residue Q254 is in hydrogen bonding distance to the γ-phosphate group of ATP, the E224 and a water molecule at the nucleotide binding pocket (Subramanya et al., 1996; Dillingham et al., 1999). The Q254 residue directly contacts the γ-phosphate of ATP, and the E224 forms hydrogen bonds with two water molecules that are hydrogen bonded to γ-phosphate oxygen atoms (Dillingham et al., 1999). Residue E224 is in an ideal position for activating the nucleophilic water molecule during hydrolysis. Mutations in residue Q254 alter the coupling between ATPase and helicase activities, and a transient interaction of Q254 with the γ-phosphate of ATP modifies the protein DNA binding site. Same mutations in residue Q254 are toxic for *E. coli* cells (Dillingham et al., 1999), and the untargeted E224V mutation might overcome such toxicity. The plasmid-borne *pcrA* Q254A (E224V) gene was also tested.

Monomeric plasmid DNA is inactive for transformation, but if it shares homology with recipient is activated upon interaction with the homologous region in the chromosome *via* RecA-mediated gene conversion, and the information present in the plasmid is transferred to the host chromosome [see Canosi et al. (1981)]. Monomeric DNA of plasmid-borne *pcrA* T65I (pCB1225), *pcrA* K37A (pCB1133), or *pcrA* Q254A (E224V) (pCB1119) was used to transform *B. subtilis* BG214 cells with selection for the plasmid marker (Table 1). Transformants carrying the established low copy unstable pCB1225, pCB1133, or pCB1119 plasmids were grown in the absence of selective pressure, and the plasmid-less segregants were subjected to nucleotide sequence analysis. We confirmed that in all transformants that lost pCB1225, the *pcrA* T65I gene had replaced the chromosomal *wt pcrA* gene by gene conversion (Petit et al., 1998). In only ~20% of the transformants that lost pCB1119, *pcrA* Q254A (E224V) mutant gene had replaced the *wt* gene, but all sequenced *pcrA* Q254A clones also contained the unselected E224V mutation. However, in all transformants that loss pCB1133, nucleotide sequence analyses revealed the presence of the *wt* gene, thus we have failed in the attempt to observe the replacement of the *wt* gene by the *pcrA* K37A mutant gene, suggesting that the K37A mutation, which impairs ATP binding, renders cells non-viable. This failure, however, could also be attributed to different reasons, for example that the mutation indirectly impacts in the expression of the downstream essential *ligA* gene (http://www.subtiwiki.uni-goettingen.de). Since the PcrAH^−^_*Sau*_ mutant variant can promote disassembly of heterologous RecA_*Eco*_ from ssDNA (Anand et al., 2007), but PcrAH^−^ fails to overcome RTCs, and depletion of *wt* PcrA in a background expressing PcrAH^−^ renders cell inviable (Merrikh et al., 2015), we have dropped the plasmid segregation approach and moved our analysis to the condition in which PcrA is selectively depleted by the *pcrA*-*ssrA sspB* degron (*pcrA*_T_) strain (Merrikh et al., 2015) to answer these puzzling observations.

### PcrA Depletion Inviability Requires RecJ and RecO, but Not RecQ, RarA, RecU, RecX, or AddAB

First, to confirm the reduction of cell survival following PcrA depletion, the *pcrA*-*ssrA sspB* degron (*pcrA*_T_) strain (Table 1) was exponentially grown to an OD_560_ = 0.4 (at 37° C) in LB medium and then, appropriate dilutions were plated on LB agar plates lacking or containing 500 μM IPTG (Materials and methods). In the absence of IPTG, the viability of the *pcrA*-*ssrA sspB* (*pcrA*_T_) degron strain was slightly compromised (1.3-fold) (Figure 1A, gray bar [- IPTG condition]) under the experimental conditions used (see below), perhaps due to noise introduced by *sspB gene* expression (see below). The plating efficiency of the *pcrA*_T_ strain was reduced ~5,000-fold upon plating onto LB agar plates containing IPTG when compared to the *pcrA*^+^ control (*rec*^+^) (Figure 1A, yellow bar [+ IPTG condition]). This is consistent with the earlier observation that *pcrA*_T_ cell viability was reduced by >1,000-fold when plated onto 100 μM IPTG-containing plates (Merrikh et al., 2015). We have observed a linear decrease in the number of viable cells with increasing IPTG concentration, but it saturates above 500 μM IPTG (data not shown). Upon IPTG addition the expression of the SspB adaptor increases, and SspB interacts with the SsrA moiety of the PcrA-SsrA protein to deliver it to the ClpXP protease for PcrA degradation [see Keiler et al. (1996), Griffith and Grossman (2008)]. It is worth mentioning that no fitness cost to *B. subtilis* cells was observed at IPTG concentrations as high as 5 mM IPTG. After 15 min of IPTG addition to the *pcrA*_T_ degron strain, 60 to 90% of PcrA is degraded (Merrikh et al., 2015).

To gain insight into PcrA contribution to repair-by-recombination, the *pcrA*_T_ degron strain was exposed to limiting H_2_O_2_ (0.2 mM) or MMS (1.3 mM) concentrations. The survival of *pcrA*-*ssrA sspB* cells in the presence of 500 μM IPTG and 0.2 mM H_2_O_2_ (blue bar [+ IPTG and H_2_O_2_]) or 1.3 mM MMS (green bar [+ IPTG and MMS]) was significantly decreased (by ~12- and ~40-fold, respectively) when compared to the absence of H_2_O_2_ or MMS (Figures 1A,B), suggesting that depletion of PcrA renders cells sensitive to both DNA damaging agents.

To elucidate whether PcrA prevents unscheduled RecA loading or dismantles its cognate recombinase assembled at or behind a stalled fork and which function(s) may counteract the PcrA antirecombinase activity, null mutants in presynaptic functions, namely end resection (Δ*addAB*, Δ*recJ*, Δ*recQ*), RecA mediation (*recO*16), and positive (Δ*rarA*) or negative (Δ*recX*, Δ*recU*) modulators (Table S1), were assessed in the *pcrA*-*ssrA sspB* (*pcrA*_T_) context (Table 1) (Sanchez et al., 2006; Cárdenas et al., 2012; Romero et al., 2020).

*B. subtilis* Δ*pcrA* lethality is suppressed in the *recL*16 background (Petit and Ehrlich, 2002). Before testing the causes of the suppression of *B. subtilis* Δ*pcrA* lethality in the *recL*16 context, we must understand the function(s) impaired in the *recL*16 strain. As described in Supplementary Material, Annex 1, the *B. subtilis* mutations previously designated as *recL*16 actually map to the *recO* locus (C. M, Marielle C. Gruenig, Michael M. Cox and J.C.A., to be published elsewhere). To simplify the analysis, the MMS^S^ phenotype was transferred by gene congression to competent BG214 cells. Five of the resulting MMS^S^ clones were selected for whole genome sequencing along with the isogenic BG214 *rec*^+^ isogenic strain (Supplementary Material, Annex 1). One of the MMS^S^ clones showed a TGA Opal stop triplet at codon 37 of *recO* and was designated recO16 (BG107-1 strain) and selected for further analysis (Table 1).

In the presence of IPTG, the survival of the *recO*16, Δ*addAB*, Δ*recX*, or Δ*recU* strain decreased ~12-, ~65-, ~3-, and ~7-fold upon addition of 0.2 mM H_2_O_2_ (blue bar [+ IPTG and H_2_O_2_]), and ~2.5 × 10^6^-, ~6 × 10^6^-, ~1 × 10^5^,- and ~1 × 10^4^-fold upon addition of 1.3 mM MMS (green bar [+ IPTG and MMS]), respectively (Figure 1). Cell survival when comparing the single Δ*recJ*, Δ*recQ*, and Δ*rarA* mutant strains and the *rec*^+^ strain was not statistically significant decreased under the H_2_O_2_ and MMS concentrations used in this assay (Figure 1), although cell survival was significantly decreased in the Δ*recJ* and Δ*recQ* background at higher H_2_O_2_ and MMS concentrations, and in the Δ*rarA* context at higher H_2_O_2_ concentrations (Sanchez et al., 2006; Romero et al., 2019a). No significant differences were observed when the survival of *rec*^+^ and *rec*–deficient strains in the absence of IPTG was compared to the presence of IPTG.

Next, the contribution of the absence of the AddAB helicase-nucleases complex (counterpart of RecBCD_*Eco*_), the RecJ 5′ → 3′ ssDNA exonuclease, or the RecQ DNA helicase (Table S1) in the *pcrA*_T_ context was analyzed. In *E. coli* cells, the RecBCD complex during the end resection process loads RecA onto naked ssDNA, to generate a 3'-overhang coated by RecA (Kowalczykowski, 2015). If AddAB works in a similar fashion it is expected that PcrA depletion inviability requires AddAB. The presence of IPTG in rich medium agar plates did not significantly affected cell viability of the Δ*recJ pcrA*_T_ (Figure 1A), and thus the lethality upon PcrA depletion was suppressed by inactivating *recJ*, but the colony area was ~9-fold smaller than in the absence of IPTG. In contrast, PcrA depletion was not suppressed by *addAB or* Δ*recQ* inactivation (Figure 1B). It is likely that the inviability upon PcrA depletion required the RecJ, but not the AddAB or RecQ functions. Furthermore, the viability of Δ*addAB pcrA*_T_ cells was significantly decreased (~10-fold), but not that of Δ*recQ pcrA*_T_ cells (~3-fold) when compared with the *pcrA*_T_ degron strain (Figure 1B).

Exponentially growing Δ*recJ pcrA*_T_, Δ*addAB pcrA*_T_, or Δ*recQ pcrA*_T_ cells were then plated on LB agar plates containing IPTG and H_2_O_2_ or MMS. The Δ*recJ* mutation significantly increased the survival of *pcrA*_T_ cells on plates containing IPTG and H_2_O_2_ (*P* < 0.001). Unexpectedly, cell survival decreased by ~3-fold on plates containing IPTG and MMS when compared with *pcrA*_T_ cells (Figure 1A), suggesting that addition of IPTG and MMS renders Δ*recJ pcrA*_T_ cells extremely sensitive (*P* < 0.001) when compared to the only IPTG condition (Figure 1A, yellow vs. green bar). Addition of IPTG and H_2_O_2_ or MMS to the Δ*recQ pcrA*_T_ strain did not significantly affect cell survival, when compared with *pcrA*_T_ cells (Figure 1B). Cell survival in the Δ*addAB pcrA*_T_ strain was significantly decreased (by ~150- and ~50-fold) on plates containing IPTG and H_2_O_2_ or MMS, respectively, when compared with *pcrA*_T_ cells (Figure 1B). It is likely that *pcrA* is not epistatic to *recJ* or *addAB* in response to MMS-induced DNA damage, and that PcrA is important possibly for backup pathways for single-strand gap and DSB repair.

Here, we have observed that there are different host requirements for the suppression of lethality between *E. coli* and *B. subtilis* cells impaired in end resection: first, the minimal medium synthetic lethality of *E. coli* Δ*rep* Δ*uvrD* cells is suppressed by *recJ* or *recQ* inactivation (Lestini and Michel, 2008), although lack of RecQ or RecJ provides very limited suppression of Δ*rep* Δ*uvrD* rich medium lethality (Guy et al., 2009). On the other hand, in *B. subtilis*, the lethality upon PcrA depletion is suppressed by *recJ* inactivation of cells grown in rich medium (Figure 1A) and it will be of significant interest to understand the molecular basis of the small colony size of Δ*recJ pcrA*_T_ upon PcrA depletion. Second, the lethality upon PcrA depletion is not suppressed by *recQ* inactivation, and survival of the Δ*recQ pcrA*_T_ cells was not significantly affected and reduced in the presence of H_2_O_2_- and MMS-induced DNA damage, respectively (Figure 1B). Unlike *E. coli*, two RecQ-like enzymes (RecQ and RecS) are present in *B. subtilis* (Fernández et al., 1998). RecQ, which is 591 amino acid long, shares ~43% identity with RecS (496 amino acid in length) if the first 346 residues containing the DExH helicase domains are used for the alignment; in short, RecS lacks the zinc-finger, the winged-helix, and the RNaseD C-terminal domains (Fernández et al., 1998; Bernstein et al., 2003). We cannot rule out that the partial genetic redundancy, exerted by RecS and RecQ, might mask the phenotype. However, since the viability of the Δ*recJ pcrA*_T_ or Δ*recQ pcrA*_T_ strain in the presence of IPTG and MMS was similar (Figures 1A,B), we have to assume that PcrA depletion and Δ*recJ* or Δ*recQ* inactivation might have different host requirements.

Next, the contribution of the *recO*16 mutation in the survival of the *recO*16 *pcrA*_T_ strain was evaluated. The presence of IPTG did not significantly affect cell viability of the *recO*16 *pcrA*_T_ strain grown in rich medium (Figure 1A). This is in good agreement with the observation that *pcrA* inactivation inviability requires RecO when grown in synthetic minimal medium (Petit and Ehrlich, 2002). To test whether PcrA works in a similar or different pathway than RecO, exponentially growing *recO*16 *pcrA*_T_ cells were plated on LB agar plates containing 500 μM IPTG and H_2_O_2_ or MMS. The survival of the *recO*16 *pcrA*_T_ strain significantly increased (>3,500-fold), when compared to the *pcrA*_T_ strain upon plating in IPTG and H_2_O_2_ containing plates. Unexpectedly, the survival of the *recO*16 *pcrA*_T_ strain was significantly decreased (>30-fold) when compared to the *pcrA*_T_ strain on plates containing IPTG and MMS (Figure 1A). Addition of IPTG and MMS rendered *recO*16 *pcrA*_T_ cells extremely sensitive (*P* < 0.001) when compared to just the addition of IPTG alone (Figure 1A, yellow vs. green bar). RecO has two activities: to load RecA onto SsbA-coated ssDNA in concert with RecR and to mediate DNA strand annealing independently of RecR (Kidane et al., 2004; Manfredi et al., 2008, 2010; Lenhart et al., 2014). Since Δ*pcrA* inviability requires RecO and RecR (Petit and Ehrlich, 2002), we assumed that the inactivation of *recO* compromises RecA loading at or behind a stalled fork, and thereby avoids unscheduled or unwanted RecA-mediated recombination during DNA replication in the context of PcrA depletion.

The contribution of mutants in the modulation of RecA filament growth in the inviability of PcrA depleted cells was also assessed. RarA has at least two activities: to control the loading of pre-primosomal proteins at a stalled fork and to positively modulate RecA filament growth (Carrasco et al., 2018; Romero et al., 2019a, 2020). PcrA depletion lethality was not suppressed by *rarA* inactivation (Figure 1B). After IPTG addition, the viability of PcrA depleted cells was significantly decreased (by ~7-fold) in the Δ*rarA pcrA*_T_ context when compared with *pcrA*_T_ cells (Figure 1B).

The absence of RarA renders cells sensitive to H_2_O_2_, but not to MMS (Romero et al., 2019a). Cell survival in the Δ*rarA pcrA*_T_ background was not significantly reduced on plates containing IPTG and H_2_O_2_ or MMS, when compared to the *pcrA*_T_ strain (Figure 1B). However, this observation is not consistent with the proposal that in *E. coli*, UvrD prevents RecA binding to ssDNA, possibly by counteracting RarA (Lestini and Michel, 2007).

RecX is a negative modulator of RecA filament growth (Cárdenas et al., 2012; Le et al., 2017), whereas RecU has two activities: to negatively modulate RecA filament growth and to cleave Holliday junctions (HJs) at a cognate site in concert with the RuvAB branch migration translocase (Ayora et al., 2004; Carrasco et al., 2005; Cárdenas et al., 2012; Cañas et al., 2014; Serrano et al., 2018). The absence of RecX or RecU did not restore viability of PcrA depleted cells grown in rich medium agar plates containing IPTG. The viability of PcrA depleted cells was significantly reduced (by ~12- and ~13-fold) in the Δ*recX pcrA*_T_ or Δ*recU pcrA*_T_ context, respectively, when compared to the *pcrA*_T_ strain (Figure 1B). The presence of IPTG and H_2_O_2_ or MMS strongly reduced cell survival in the Δ*recX pcrA*_T_ (~30- and ~40-fold) and Δ*recU pcrA*_T_ (~13- and ~40-fold) backgrounds, respectively, when compared to the *pcrA*_T_ strain (Figure 1B), suggesting that the *pcrA* gene is likely not epistatic to *recX* or *recU* in response to non-bulky DNA lesions of oxidative nature. In *E. coli*, Δ*uvrD* Δ*ruvC* (counterpart of *B. subtilis* RecU) cells are inviable (Magner et al., 2007).

### PcrA Depletion Inviability Requires RecA

Rich medium synthetic lethality of *E. coli* Δ*uvrD* Δ*rep* cells is not suppressed by *recA* inactivation (Veaute et al., 2005; Guy et al., 2009). Similarly, *B. subtilis pcrA* lethality is not suppressed when the leaky *recA*1 (formerly termed *recE*1) mutation is moved into the background (Petit and Ehrlich, 2002), but the RecA1 activities present in the background may mask the outcome (e.g., competent *recA*1 cells are marginally affected, whereas null *recA* cells are blocked in chromosomal transformation) (Alonso et al., 1988). To address whether *recA* inactivation suppresses cell inviability upon PcrA depletion, a *recA* null mutant allele (Δ*recA*) was moved, *via* SPP1-mediated generalized transduction, onto the *pcrA*_T_ strain (see Table 1). Absence of RecA reduced survival of the parental strain by ~80-fold in response to H_2_O_2_, and strongly reduced cell survival (~6 ×10^6^-fold) in response to MMS, when compared to the *wt* strain (Figure 1A).

The lethality of PcrA depleted cells was significantly suppressed (*P* < 0.001) by *recA* inactivation upon IPTG addition to rich LB agar plates (Figure 1A), but the colony area was ~17-fold smaller than in the absence of IPTG. Addition of IPTG and H_2_O_2_ or MMS did not significantly reduce cell survival in the Δ*recA pcrA*_T_ background when compared to the Δ*recA* strain (Figure 1A, blue vs. green bar). To re-evaluate the results in the Δ*recA pcrA*_T_ background, the Δ*recA pcrA*_T_ strain was reconstructed, and similar results were observed. The apparent contradiction between PcrA depleted cells in the Δ*recA* context (Figure 1A) with the Δ*pcrA recA*1 condition (Petit and Ehrlich, 2002), can be attributed either to the leaky *recA*1 mutation or to background differences, since there are extrachromosomal elements (as the conjugative element ICE*Bs1* and the prophage SPβ) in the Δ*pcrA recA*1 context (Petit and Ehrlich, 2002) that are absent in the Δ*recA pcrA*_T_ background (see Table 1).

It is likely, therefore, that: (i) inviability upon PcrA depletion could not be associated with the inability to promote auto-proteolysis of the transcriptional repressor LexA, because inactivation of *recA* prevents SOS induction, but suppresses *pcrA*_T_ lethality in the presence of IPTG (Figure 1A); and (ii) PcrA depletion inviability requires RecA, and *pcrA* is epistatic to *recA* in response to non-bulky DNA lesions of oxidative nature.

### PcrA Depletion Leads to Unsegregated Chromosomes

As previously proposed for eukaryotic Srs2 (Marini and Krejci, 2010), the role of PcrA might be to prevent the accumulation of crossovers (CO) by promoting synthesis-dependent strand annealing (SDSA), leading to the exclusive accumulation of non-crossover (NCO) products. In circular chromosomes, the outcome of CO and NCO will be a dimeric or two monomeric chromosomes, respectively. Dimeric chromosomes are deleterious and need to be processed before cell division. To test whether PcrA prevents CO accumulation, the chromosome segregation of PcrA depleted cells was studied.

Branched DNA structures can be processed by different pathways. First, the RecU HJ resolvase, in concert with the RuvAB translocase, cleaves the outside or the inside strands of a HJ, followed by religation to produce CO and NCO products, respectively (Carrasco et al., 2004; Cañas et al., 2014). Second, a HJ can be dissolved by the RecQ helicase in concert with a Type I DNA topoisomerase to produce NCO products (Kowalczykowski, 2015). Finally, PcrA may dismantle RecA nucleoprotein filaments from branched structures and may promote SDSA by unwinding the elongated invading strand, a step that is followed by annealing to the ssDNA of the other end of the break, an event associated with NCO products (Marini and Krejci, 2010).

**Figure 2:**
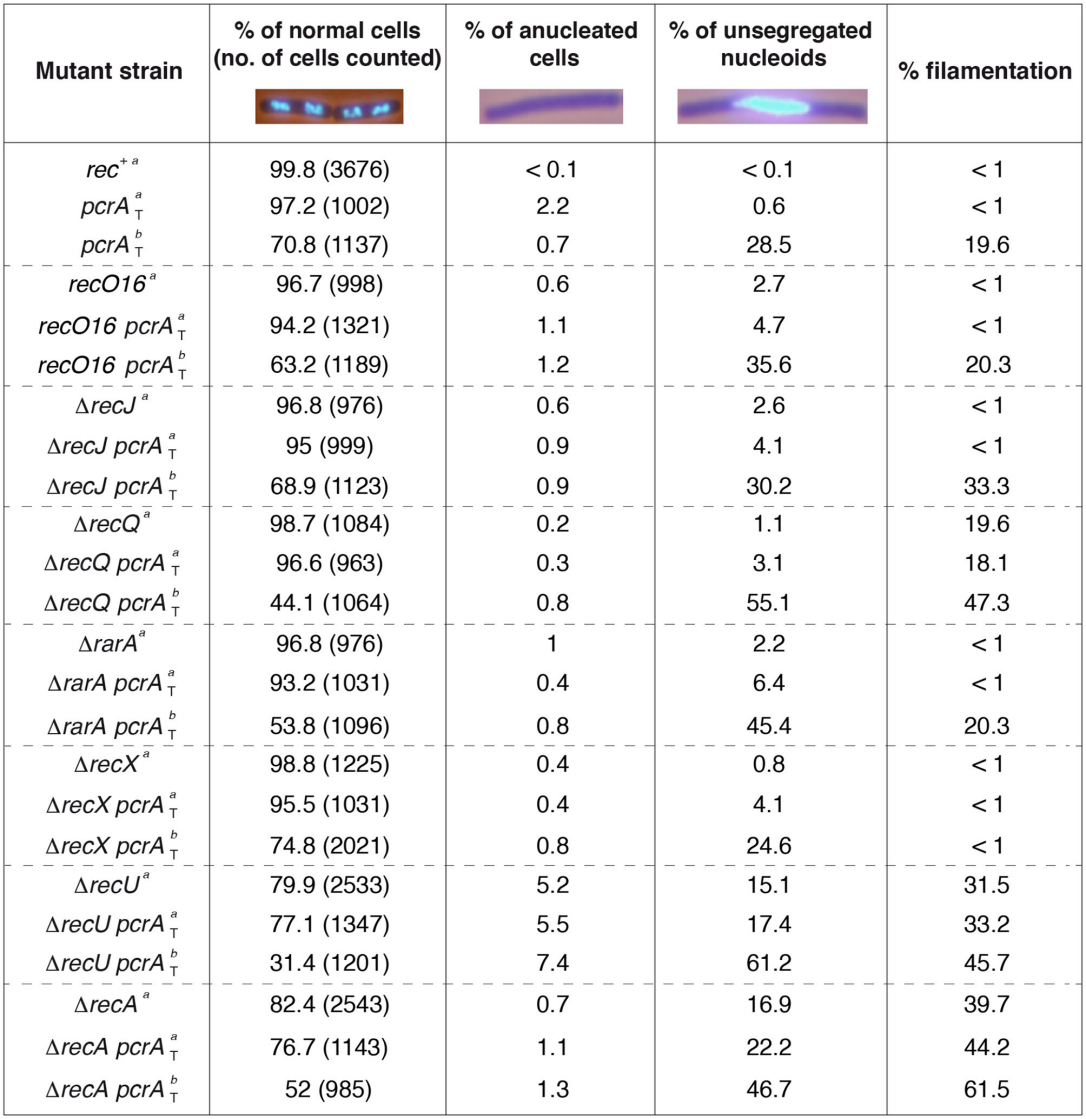
Chromosome segregation in the absence of presynaptic or synaptic functions. Cells were grown in LB medium to OD_560_ = 0.2; after 60 min, cells were harvested, prepared for DAPI DNA-fluorescence microscopy, and the percentage of anucleate and unsegregated nucleoids determined (condition *a*). In parallel, at OD_560_ = 0.2, IPTG (500 μM) was added and after 60 min, cells were harvested, DAPI-stained, and the percentage of anucleate and unsegregated nucleoids determined (condition *b*). Representative fluorescent images of two dividing DAPI-treated cells (DNA stain, light blue) are shown. The pictures are taken at the same amplification, two none separated cells (four nucleoids) are presented under normal conditions. The mean of at least three independent experiments is shown.

To evaluate whether PcrA depletion provokes a chromosomal segregation defect, the nucleoid (supercoiled and compacted chromosome bound by proteins) of the *pcrA*_T_, *recO*16 *pcrA*_T_, Δ*recJ pcrA*_T_, Δ*recQ pcrA*_T_, Δ*rarA pcrA*_T_, Δ*recX pcrA*_T_, Δ*recU pcrA*_T_, or Δ*recA pcrA*_T_ cells was DAPI-stained and analyzed by fluorescence microscopy. As controls we have used the Δ*recU* and Δ*recA* strains. In the absence of any external DNA damage and at mid-exponential phase, Δ*recU* or Δ*recA* mutations reduce the number of CFUs by ~5- and ~10-fold (Figure 1), and ~30 and ~40% of cells are filamented, respectively (Figure 2) (Carrasco et al., 2004), suggesting that in the absence of RecU or RecA, a cell subpopulation undergoes a death-by-recombination phenotype.

Previously it has been shown that after 15 min (37°C) of 100 μM IPTG addition to *B. subtilis* cells 60 to 90% of PcrA is degraded (Merrikh et al., 2015). In this study, cells were grown in rich medium under unperturbed conditions until they reached OD_560_ = 0.2 (37°C). IPTG (500 μM) was added to half of the culture, and both cultures were incubated (60 min, 37°C) before harvesting, fixing and staining the cells with DAPI. During vegetative growth, net accumulation of anucleated cells, unsegregated chromosomes and filamented cells was rare in *rec*^+^ cells in the absence (Figure 2) or presence of IPTG (data not shown). In this scenario, cells displayed an average length of 4–6 μm, and exhibited a bimodal distribution of nucleoid positioning with ~35% of total cells having two nucleoids, and ~65% of total cells containing only one nucleoid with about twice the fluorescence signal as judged by eye (Carrasco et al., 2004). This suggests that the former class were replicated cells with segregated chromosomes and the latter were replicated cells with yet-unsegregated chromosomes.

In the absence of IPTG, ~97% of *pcrA*-*ssrA* cells appeared normal compared to the *rec*^+^ control (~100%) (Figure 2). Absence of DAPI staining (anucleated cells) significantly increased (by ~20-fold) and the fraction of cells with aberrant chromosomes by ~6-fold when compared to the *rec*^+^ control, whereas upon IPTG addition absence of DAPI staining was significantly decreased (Figure 2), suggesting that the *pcrA*-*ssrA* fusion or noise from *sspB* gene expression affects chromosomal segregation of unperturbed exponentially growing *pcrA*_T_ cells. In the presence of IPTG, PcrA dropped, the proportion of cells with an incompletely separated nucleoid or aberrant chromosomes increased by ~50-fold when compared to the condition without IPTG, and the average cell length was >8 μm in ~20% of total *pcrA*_T_ cells (Figure 2). Since those elongated cells contained a single nucleoid it was assumed that they were filamented cells. It is likely that, upon PcrA depletion, NCO shifted toward CO products. In other words, PcrA might suppress COs or might directly contribute to the formation of NCO products, as was shown for its yeast homolog Srs2 (Marini and Krejci, 2010).

In the absence of IPTG, the proportion of anucleated cells and cells with aberrant chromosomes was marginally affected in the *recO*16 *pcrA*_T_, Δ*recJ pcrA*_T_, and Δ*recA pcrA*_T_ strains when compared to the single mutant strain, but in the Δ*recA* or Δ*recA pcrA*_T_ condition 40–45% of total cells were filamented (Figure 2). In the presence of IPTG, the absence of DAPI staining and the proportion of unsegregated nucleoids were not significantly affected (<2-fold) when compared to the parental control (*rec*^+^ *pcrA*_T_) strain (Figure 2). Likewise, in the *recO*16 *pcrA*_T_, Δ*recJ pcrA*_T_, and Δ*recA pcrA*_T_ conditions a significant proportion of cells formed filaments, with <2-fold increase in the proportion of filamented cells when compared to the most affected parental strain (Figure 2). It is likely that the PcrA pro-SDSA function requires RecJ, RecO, or RecA.

The remaining strains were classified into three different classes. First, for RarA and RecQ, which have two activities each (see above): inactivation of *recQ* or *rarA* revealed a marginal chromosome segregation defect, but ~20% of total Δ*recQ* cells formed filaments (Figure 2). In the presence of IPTG, the proportion of unsegregated nucleoids significantly increased in Δ*recQ pcrA*_T_ or Δ*rarA pcrA*_T_ when compared to the *pcrA*_T_ control, but absence of DAPI staining was not significantly affected (Figure 2). Upon PcrA depletion the proportion of filamentous cells was similar in *pcrA*_T_ and Δ*rarA pcrA*_T_ cells, but significantly increased in the Δ*recQ pcrA*_T_ context. Second, RecX negatively modulates RecA filament growth (Cárdenas et al., 2012; Le et al., 2017). In the presence of IPTG, the proportion of unsegregated nucleoids in Δ*recX pcrA*_T_ was similar to the *pcrA*_T_ control, but counteracted the formation of filamented cells (Figure 2). Third, for RecU, which has two activities (see above): in the absence of RecU, ~5% of total cells were anucleated as previously described (Carrasco et al., 2004), suggesting that cell division occurred in regions that had not received a nucleoid, and unsegregated nucleoids accounted up to ~15% of total cells. In the presence of IPTG, most of Δ*recU pcrA*_T_ cells (~60%) had unsegregated nucleoids, and ~45% of cells were present as “filaments” (Figure 2), suggesting that PcrA may promote SDSA prior to the formation of a double-HJ that can be resolved to NCO and CO by the RecU HJ resolvase in concert with the RuvAB branch migration translocase (Ayora et al., 2004; Cañas et al., 2014).

Altogether, the data presented in Figures 1, 2 revealed certain paradoxes. First, the inviability of PcrA depletion requires RecJ, RecO, and RecA, but under these conditions a chromosomal segregation defect was observed, suggesting that PcrA processes branched DNA structures formed at replication or at replication-transcription stalled forks, but with the help of accessory proteins (e.g., RecJ, RecO) a formed RecA nucleoprotein filament may be dismantled by PcrA. Alternatively, PcrA removes proteins bound to stalled forks to indirectly allow the formation of branched structures. Second, PcrA depletion halts cell proliferation, initiates accumulation of unprocessed branched intermediates, and additively reduces repair-by-recombination in the Δ*recQ* or Δ*rarA* context. Finally, PcrA depletion exacerbates the segregation defect of Δ*recU* cells, with only ~30% having normal chromosomal segregation, and in the absence of both negative RecA modulators there is a synergistic repair-by-recombination defect.

### PcrA Inviability Requires Mfd, but Not RecD2, HelD, HepA, or YwqA

Enzymes of the UvrD family of translocases provide different solutions to cope with a replicative stress and/or RTCs. UvrD_*Eco*_ and PcrA can interact with and backtrack RNAP *in vivo* and *in vitro*, that is a crucial step for minimizing RTCs and for the repair of lesions occluded by a stalled RNAP, which become a major obstacle to DNA replication (Epshtein et al., 2014). Other DNA helicases/translocases of SF1, namely HelD and RecD2, and of SF2, such as Mfd, HepA (YqhH), and YwqA, also interact with RNAP *via* a conserved domain (Table S2) (Muzzin et al., 1998; Sukhodolets et al., 2001; Deaconescu et al., 2006; Shaw et al., 2008; Guy et al., 2009; Boubakri et al., 2010; Jin et al., 2011; Epshtein et al., 2014; Wiedermannova et al., 2014; Liu et al., 2015; Sanders et al., 2017; Le et al., 2018). These enzymes have been also implicated: (i) in preventing or mitigating the impact of protein-DNA complexes or spontaneous non-bulky DNA lesions of oxidative nature that halt transcription or replication, (ii) in avoiding the conflicts generated by the collision between the replication and transcription machineries, and (iii) in promoting RNAP recycling, sliding backward along the template (backtracking or retreating) or RNAP removal, or when replication forks are arrested by the formation of R-loops (Ayora et al., 1996; Komissarova and Kashlev, 1997; Sukhodolets et al., 2001; Trautinger et al., 2005; Deaconescu et al., 2006; Guy et al., 2009; Yawn et al., 2009; Boubakri et al., 2010; Gupta et al., 2013; Bruning et al., 2014; Wiedermannova et al., 2014; Merrikh et al., 2015; Sanders et al., 2017; Ho et al., 2018; Le et al., 2018).

**Figure 3:**
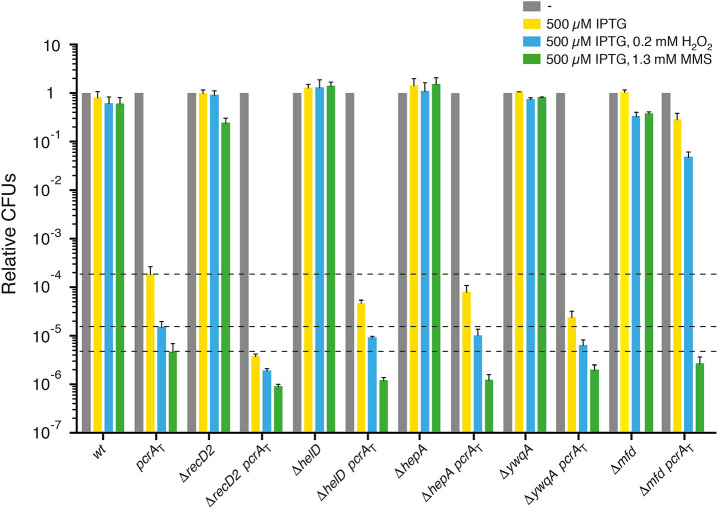
PcrA lethality is suppressed by *mfd* inactivation, but not by *recD2, helD, hepA, ywqA* inactivation. Log phase cultures of *wt*, single or double mutant strains were diluted and plated on LB agar containing 500 μM IPTG (yellow bars) or lacking it (gray bars). Lethality assays showing cell viability upon PcrA depletion in *recD2, helD, hepA, ywqA*, or *mfd* cells. Log phase cultures of indicated strains were diluted and plated on LB agar containing IPTG and 0.2 mM H_2_O_2_ (blue bars) or IPTG and 1.3 mM MMS (green bars). Experiments were performed at least four times. The dotted lines mark the survival rate upon PcrA depletion. Data are shown as mean fractional survival ± SEM.

To study whether the lack of PcrA destabilization of transcription complexes is the primary cause of inviability, the Δ*helD pcrA*_T_, Δ*recD2 pcrA*_T_, Δ*hepA pcrA*_T_, Δ*ywqA pcrA*_T_, and Δ*mfd pcrA*_T_ strains were constructed as described (Materials and methods). PcrA depletion, which decreases cell survival in the Δ*recD*2 context (Torres et al., 2017), was used as control. Except in the Δ*mfd* strain, the H_2_O_2_ or MMS concentrations used were not sufficient to reveal a reduced viability phenotype (Figure 3).

Upon addition of IPTG, the lethality observed following PcrA depletion was not suppressed by *recD*2 inactivation (Figure 3). In the presence of IPTG, the viability was significantly decreased (by ~25-fold) in Δ*recD*2 *pcrA*_T_ when compared to the *pcrA*_T_ strain. Addition of both IPTG and H_2_O_2_ or MMS significantly reduced cell survival (by ~8- and ~5-fold, respectively) in the Δ*recD*2 *pcrA*_T_ cells when compared to the *pcrA*_T_ strain (Figure 3), suggesting that *pcrA* is not epistatic to *recD*2 in response to H_2_O_2_- or MMS-induced DNA damage.

Inactivation of the *helD* helicase partially suppresses the DNA repair defect of *recF*15, Δ*recO*, and Δ*recR* cells (Carrasco et al., 2001). The absence of HelD did not suppress cell inviabilily upon PcrA depletion; in this background, cell viability was not significantly decreased when compared to the *pcrA*_T_ control upon addition of IPTG (Figure 3, yellow bar [+ IPTG]). Upon addition of IPTG and MMS, cell survival was significantly decreased (by ~4-fold), when compared to the *pcrA*_T_ strain (Figure 3, green bar [+ IPTG and MMS]). In contrast, in the absence of *E. coli* HelD and UvrD cells remain recombination proficient and apparently are as capable of repairing MMS-induced DNA damage as the *wt* control (Mendonca et al., 1993). Addition of IPTG and H_2_O_2_ marginally decreased cell survival (by ~2-fold) (Figure 3, blue bar [+ IPTG and H_2_O_2_]).

The absence of HepA or YwqA did not suppress cell inviability upon PcrA depletion (Figure 3). Cell viability was significantly decreased (by ~8-fold) in the Δ*ywqA pcrA*_T_ when compared to the *pcrA*_T_ control, but not in the Δ*hepA pcrA*_T_ (decreased by ~3-fold) when compared to the *pcrA*_T_ control (Figure 3).

Addition of IPTG and H_2_O_2_ did not significantly reduce survival in Δ*hepA pcrA*_T_ or Δ*ywqA pcrA*_T_ cells when compared to the *pcrA*_T_ strain (Figure 3). The presence of IPTG and MMS significantly reduced cell survival in the Δ*hepA pcrA*_T_, but marginally reduced cell survival in Δ*ywqA pcrA*_T_ cells when compared to the *pcrA*_T_ strain (Figure 3). These data altogether suggest that decreasing the probability of backtracking events contributes to maintaining genome stability, but not to suppress the lethality of PcrA depleted cells. Unlike in *E. coli* cells (Shaw et al., 2008; Jin et al., 2011; Liu et al., 2015), we have little information about how the *B. subtilis* HepA or YwqA ATPase propels backward translocation of the RNAP along the DNA template or release a sequestered RNAP.

*E. coli* cells lacking Mfd show a weak sensitivity to UV irradiation (Witkin, 1969). *E. coli* Mfd recruits UvrA to the site of a roadblock that stalls RNAP. This activity is crucial for the recognition and removal of a stalled RNAP, but Mfd is subsequently displaced by UvrB to initiate transcription coupled repair (Selby and Sancar, 1993, 1994; Ayora et al., 1996; Manelyte et al., 2010; Epshtein, 2015; Ho et al., 2018, 2020; Le et al., 2018). In contrast, inactivation of *B. subtilis mfd* renders cells significantly sensitive to the UV mimetic 4-nitroquinoline-1-oxide and also to oxidative non-bulky lesions as those generated by exposure to MMS (Ayora et al., 1996). Our results suggested that Mfd is also required in *B. subtilis* cells to repair non-bulky lesions (Figure 3). It is likely that in *B. subtilis*: (i) bulky and non-bulky DNA lesions stall RNAP; and (ii) Mfd interacts with and dislodges RNAP from the damaged DNA template.

Upon addition of IPTG, inactivation of *mfd* significantly suppressed (*P* < 0.001) the lethality induced by PcrA depletion (Figure 3), but the colonies were minute and with an area ~19-fold smaller than in the absence of IPTG, suggesting that Mfd and PcrA play a crucial role in response to a replicative stress. The Δ*mfd* mutation significantly suppressed the sensitivity of Δ*mfd pcrA*_T_ cells to H_2_O_2_ (addition of both IPTG and H_2_O_2_), but Δ*mfd pcrA*_T_ cells showed a non-significant decrease when compared to the *pcrA*_T_ strain in the presence of both IPTG and MMS (Figure 3). It is likely that the *pcrA* gene is epistatic to *mfd* in response to MMS-induced DNA lesions. Inactivation of *E. coli mfd* partially suppresses the sensitivity to UV irradiation in the *uvrD* context (Epshtein et al., 2014).

## Conclusions

We show that PcrA depletion lethality is suppressed by *recJ, recO*16, or *recA* inactivation, but not by *addAB, recQ, rarA, recX*, or *recU* inactivation when cells are grown in rich medium (Figures 1A,B). These data suggest that PcrA depleted cells primarily die due to their inability to resuscitate replisomes blocked by a RecA-ssDNA complex. Indeed, RecO loads RecA onto SsbA-coated ssDNA and a RecA nucleoprotein filament downregulates initiation of PriA-dependent DNA replication *in vitro* (Vlasic et al., 2014), and PcrA depletion inviability requires RecA for replication re-start (Million-Weaver et al., 2015). This is consistent with the observation that PcrA depletion inviability also requires RecO, which loads RecA onto ssDNA (Carrasco et al., 2015), but not AddAB. In a minimal synthetic medium, the *pcrA* inactivation lethality is also suppressed by inactivation of the *recO* or *recR* positive mediators or a leaky mutation in the positive *recF*17 modulator (Petit and Ehrlich, 2002).

In *E. coli* cells, the synthetic lethality of *uvrD* and *rep* mutations is partially suppressed by *recJ* or *recQ* inactivation in minimal medium (Lestini and Michel, 2008). We can envision that the discrepancies observed between *E. coli* and *B. subtilis* cells are related to genetic differences between these genetically distant bacteria. First, *B. subtilis* cells have two RecQ-like helicases, RecQ and RecS, with the latter potentially masking the outcome, whereas *E. coli* has only RecQ. Second, *E. coli* cells have two proteins (UvrD and RecX) to actively dismantle a RecA nucleoprotein filament (Petrova et al., 2015; Le et al., 2017), whereas *B. subtilis* cells have four different proteins (PcrA, RecX, RecU, and RecD2) to do this job (Anand et al., 2007; Le et al., 2017; Torres et al., 2017; Serrano et al., 2018). PcrA was also found to be necessary to survive DNA damage. The *pcrA* gene is not epistatic to genes involved in end resection (*addAB, recJ, recQ*), RecA mediators (*recO*16), or negative RecA modulators (*recX, recU*) in response to MMS- or H_2_O_2_-induced DNA damage, but it is epistatic to the *recA* gene, suggesting that PcrA also contributes to repair-by-recombination *via* poorly understood mechanisms. The role of the positive RecA modulator RarA upon PcrA depletion requires further studies.

As it has been seen previously for Srs2 (Marini and Krejci, 2010), PcrA might play a putative role in promoting SDSA, which does not entail the generation of COs. Depletion of PcrA leads to additive, in *recJ, recO* and *recA*, and to synergic accumulation of unsegregated chromosomes in *recQ, rarA recU* backgrounds. The dual activities of these proteins (see above) mask the interpretation of our results.

Finally, we show that PcrA depletion lethality is suppressed by *mfd* inactivation, but not by *recD*2, *helD, hepA*, or *ywqA* inactivation (Figure 3). We show that *pcrA* is not epistatic to *recD*2, *helD*, or *hepA* in response to non-bulky DNA damage, but it is epistatic to *mfd*. The role of the poorly characterized YwqA ATPase upon PcrA depletion requires further studies.

PcrA and Mfd might act on RTCs, both dependent (Ayora et al., 1996) and independently of the nucleotide excision repair pathway (Figure 3). The PcrA and Mfd translocases physically interact with stalled RNAPs at lesions on the DNA template. PcrA is a pro-backtracking factor by promoting forward RNAP translocation, and Mfd might be an anti-backtracking that dislodges a stalled RNAP, as previously postulated for the isolated protein *in vitro* (Selby and Sancar, 1993; Ayora et al., 1996; Park et al., 2002; Deaconescu et al., 2006; Epshtein et al., 2014; Sanders et al., 2017; Ho et al., 2018; Le et al., 2018). It is likely that when damaged template bases interfere with RNAP progression, it backtracks and becomes transiently arrested. Under this condition, Mfd and PcrA, which interact with UvrA and UvrB, respectively, are crucial factors involved in mitigating RTCs in the presence of DNA lesions that are or not targeted by transcription coupled repair. Thus, we propose that PcrA is crucial to remove a stalled RNAP that would otherwise hinder DNA replication even in the presence of DNA lesions that are not targeted by transcription coupled repair. In other words, in the absence of PcrA, RNAP may not be evicted from the damage site by Mfd, leading to a harmful genotoxic stress that induces lethality. PcrA allows genome duplication to occur concurrently with other essential DNA transactions (replication, transcription, repair, segregation). Partial PcrA depletion sensitizes cells to severe DNA transactions and its major role is to work in concert with recombination and repair proteins at stalled DNAP/RNAP complexes to facilitate replication progression beyond the conflict point.

## Data Availability Statement

The raw data supporting the conclusions of this article will be made available by the authors, without undue reservation.

## Author Contributions

MM, RT, and JA designed the experiments and drafted the manuscript. MM, RT, CM, JR-M, and GS performed the experiments. JA coordinated the research and wrote the manuscript. MM, RT, CM, JR-M, GS, and JA interpreted the data. All authors contributed to the article and approved the submitted version.

## Conflict of Interest

The authors declare that the research was conducted in the absence of any commercial or financial relationships that could be construed as a potential conflict of interest.
