# Supplementary material for: Bacillus subtilis PcrA Couples DNA Replication, Transcription, Recombination and Segregation
Source: Front Mol Biosci. 2020 Jul 21;7:140. doi: 10.3389/fmolb.2020.00140 (PMC7385302; doi:10.3389/fmolb.2020.00140)
Supplement: Supplementary file 1 [file Data_Sheet_1.docx]

*Bacillus subtilis* PcrA couples DNA replication, transcription, recombination and segregation

**María Moreno-del Alamo^1^,** **Rubén Torres^1^, Candela Manfredi^1,§^, José A. Ruiz-Masó^2^, Gloria del Solar^2^, and Juan Carlos Alonso^1,*^**

^1^Department of Microbial Biotechnology, Centro Nacional de Biotecnología, CNB-CSIC, 28049 Madrid, Spain

^2^Centro de Investigaciones Biológicas Margarita Salas, CIB-CSIC, 28040 Madrid, Spain

## *Annex 1*. The *recL*16 mutations map in the *recO* gene

Before testing the causes of the suppression of *B. subtilis* Δ*pcrA* lethality in the *recL*16 background (1), we must understand the function(s) impaired in the *recL*16 strain. The putative RecL function, which acts before RecA, contributes to RecA filament growth (RecA*, which chaperones the LexA transcriptional repressor, facilitates its auto-cleavage, thereby activating the SOS response), thus positively controls the induction of the SOS response (2). To unravel the defect associated to the *recL*16 mutation, the mutation(s) present in the original BG107 strain (Table 1) were mapped using a custom-designed microarray and measuring hybridization of the DNA of the original BG107 test strain in comparison with the reference BG214 *rec*^+^ strain. The *B. subtilis* mutations previously designated as *recL*16 actually map to the *recO* locus (C. M, Marielle C. Gruenig, Michael M. Cox and J.C.A., to be published elsewhere). Mutations in the *hemE*, *pksL* and *deaD* genes and a synonymous mutation in the *iolG* gene were also observed in the BG107 test strain (C. M, M.C.G., M.M.C. and J.C.A., to be published elsewhere). There are, however, some phenotypic discrepancies between the *recL*16 and the absence of the *recO* gene in the Δ*recO* strain (3). It is likely, therefore, that *recL*16 is a division of labor mutant, proficient in strand annealing, but impaired as a RecA mediator.

The RecO protein has two activities: to recruit RecA onto SsbA-ssDNA complexes and to facilitate DNA strand annealing. The first activity is essential for the *in vivo* function of RecA nucleation onto SsbA-coated ssDNA (RecA*) and to activate RecA to catalyze DNA strand exchange during repair-by-recombination (2, 4-6). The second activity of RecO is crucial for single strand annealing of complementary strands, at least during natural plasmid transformation (7, 8). Both *recL*16 and Δ*recO* mutations rendered cells equally sensitive to DNA damaging agents, however the plasmid transformation efficiency is reduced ~25-fold in the Δ*recO* background, and only marginally impaired in the *recL*16 mutant strain (3).

To simplify the analysis, the *recL*16 MMS^S^ phenotype was transferred by gene congression to competent BG214 cells with primary selection for a plasmid-borne marker (Cm^R^) as described (Materials and methods). Then, by replica plating onto MMS-containing plates, five clones with the MMS^S^ phenotype were selected. Finally, we have performed nucleotide sequence analyses of five MMS^S^ clones, using genome analyzer (Illumina) data analysis of ~1 gigabase of filtered sequences from fragmented libraries, followed by whole-genome comparison in parallel with the isogenic BG214 *rec*^+^ reference strain. Three of the sequenced clones showed that the CGA triplet coding for Arg at codon 37 was replaced by the TGA Opal stop triplet and the ATG triplet at codon 122 coding for Met was replaced by the TTG (Leu) codon (M122L) as in the original BG107 test strain (C.M., M.C.G., M.M.C and J.C.A., to be published elsewhere). The fourth sequenced MMS^S^ clone only showed the TTG triplet at codon 122 (M122L), and the fifth one showed the TGA Opal stop triplet at codon 37. The last clone was renamed as *recO*16 (BG107-1 strain), and selected for further analysis (Table 1). Except for the mutation in the *recO* gene, none of the sequenced MMS^S^ clones carried mutations in any other gene when compared to the BG214 parental strain (Table 1).

**Table S1.** *B. subtilis* proteins involved in the formation of a RecA dynamic filament

| Protein name^a^ | Protein function | Interacting partner(s) | Protein activity |
| --- | --- | --- | --- |
| AddAB | End resection | AddA, AddB | Nuclease-helicase complex |
| RecJ | End resection | SsbA, RecQ, RecS | 5´→3´ ssDNA exonuclease |
| RecQ | End resection | SsbA, TopB | 3´→5´ DNA helicase |
| RecA | Recombinase | RecX, RecU | Strand invasion and exchange |
| RecO | RecA mediator | SsbA | Strand annealing, RecA loader |
| RarA | RecA modulator | SsbA | AAA^+^ ATPase |
| RecU | RecA modulator | RecA | HJ resolvase, RecA disassembly |
| RecX | RecA modulator | RecA | RecA-ssDNA disassembly |
| PcrA | RecA modulator | SsbA, UvrB, RNAP | 3´→5´ DNA helicase |
| RecD2 | RecA modulator | SsbA, RNAP | 5´→3´ DNA helicase |

^a^The references for the activities are cited in the main text.

**Table S2.** *B. subtilis* proteins involved in replication-transcription interface

| Protein name^a^ | Protein function | Interacting partner(s) | Protein activity |
| --- | --- | --- | --- |
| PcrA | Replication-transcription | SsbA, UvrB, RNAP | 3´→5´ DNA helicase |
| RecD2 | Replication-transcription | SsbA, RNAP | 5´→3´ DNA helicase |
| RarA | Replication | SsbA | AAA^+^ ATPase |
| HelD | Replication-transcription | RNAP | 3´→5´ DNA helicase |
| Mfd | Transcription coupling repair | UvrA, RpoB | Translocase |
| RapA | Replication-transcription | RNAP | ATPase |
| YwqA | Replication-transcription | RNAP | ATPase |

^a^The references for the activities are cited in the main text.

**References**

1. M. A. Petit and D. Ehrlich: Essential bacterial helicases that counteract the toxicity of recombination proteins. *EMBO J*, 21(12), 3137-47 (2002)

2. M. Gassel and J. C. Alonso: Expression of the *recE* gene during induction of the SOS response in *Bacillus subtilis* recombination-deficient strains. *Mol Microbiol*, 3(9), 1269-76 (1989)

3. S. Fernández, Y. Kobayashi, N. Ogasawara and J. C. Alonso: Analysis of the *Bacillus subtilis* recO gene: RecO forms part of the RecFLOR function. *Mol Gen Genet*, 261(3), 567-73 (1999)

4. P. P. Cárdenas, B. Carrasco, C. Defeu Soufo, C. E. César, K. Herr, M. Kaufenstein, P. L. Graumann and J. C. Alonso: RecX facilitates homologous recombination by modulating RecA activities. *PLoS Genet*, 8(12), e1003126 (2012) doi:10.1371/journal.pgen.1003126

5. B. Carrasco, T. Yadav, E. Serrano and J. C. Alonso: *Bacillus subtilis* RecO and SsbA are crucial for RecA-mediated recombinational DNA repair. *Nucleic Acids Res*, 43(12), 5984-97 (2015) doi:10.1093/nar/gkv545

6. C. Manfredi, B. Carrasco, S. Ayora and J. C. Alonso: *Bacillus subtilis* RecO nucleates RecA onto SsbA-coated single-stranded DNA. *J Biol Chem*, 283(36), 24837-47 (2008) doi:M802002200 [pii] 10.1074/jbc.M802002200

7. C. Manfredi, Y. Suzuki, T. Yadav, K. Takeyasu and J. C. Alonso: RecO-mediated DNA homology search and annealing is facilitated by SsbA. *Nucleic Acids Res*, 38(20), 6920-9 (2010) doi:gkq533 [pii] 10.1093/nar/gkq533

8. T. Yadav, B. Carrasco, A. R. Myers, N. P. George, J. L. Keck and J. C. Alonso: Genetic recombination in *Bacillus subtilis*: a division of labor between two single-strand DNA-binding proteins. *Nucleic Acids Res*, 40(12), 5546-59 (2012) doi:10.1093/nar/gks173
